# Need for NAD^+^: Focus on Striated Muscle Laminopathies

**DOI:** 10.3390/cells9102248

**Published:** 2020-10-07

**Authors:** Déborah Cardoso, Antoine Muchir

**Affiliations:** INSERM, Institute of Myology, Center of Research in Myology, Sorbonne University, 75013 Paris, France; d.cardoso@institut-myologie.org

**Keywords:** striated muscle laminopathies, NAD^+^, dilated cardiomyopathy, Emery–Dreifuss muscular dystrophy, therapies

## Abstract

Laminopathies are a heterogeneous group of rare diseases caused by genetic mutations in the *LMNA* gene, encoding A-type lamins. A-type lamins are nuclear envelope proteins which associate with B-type lamins to form the nuclear lamina, a meshwork underlying the inner nuclear envelope of differentiated cells. The laminopathies include lipodystrophies, progeroid phenotypes and striated muscle diseases. Research on striated muscle laminopathies in the recent years has provided novel perspectives on the role of the nuclear lamina and has shed light on the pathological consequences of altered nuclear lamina. The role of altered nicotinamide adenine dinucleotide (NAD^+^) in the physiopathology of striated muscle laminopathies has been recently highlighted. Here, we have summarized these findings and reviewed the current knowledge about NAD^+^ alteration in striated muscle laminopathies, providing potential therapeutic approaches.

## 1. Introduction

Laminopathies are a heterogeneous group of genetic disorders caused by mutations in *LMNA*, which encodes nuclear A-type lamins: lamins A and C. Generated by alternative splicing, lamins A and C are identical for most of their sequence but differ from their *C*-terminal domains [1]. The central rod domain of A-type lamins consists of heptad repeats, which drives the interaction between two lamin proteins, the basic structural unit of lamins assembly. The rod domain is flanked by *N*- and *C*-terminal non-α-helical domains. The *C*-terminal domain has a nuclear localization sequence (NLS) for nuclear import (sequence KKRKLE). In addition, lamin A but not lamin C has a CaaX motif (sequence CSIM), which undergoes cleavage of the last three amino acids and isoprenylation of the terminal cysteine residue. The *C*-terminal 18 amino acids of lamin A, including the isoprenyl group, is removed by proteolysis. Lamins A and C, which are members of the intermediate filaments family, dimerize and further assemble to form the nuclear lamina [2], a fibrillar meshwork contributing to cellular functions such as mechanotransduction, regulation of gene transcription and chromatin structure [3]. These intermediate filament proteins, the only intermediate filaments located within the nucleus, are expressed in most tissues.

The nuclear lamina is part of the nuclear envelope, which defines the barrier between the nucleus and cytoplasm and features inner and outer membranes separated by a perinuclear space. The nuclear envelope contains specific integral and associated proteins that form a mechanical link, called the LINC (LInker of Nucleoskeleton and Cytoskeleton) complex, tethering the nucleo- and cytoskeleton via the nuclear envelope. This LINC complex may serve as a mechanosensor, translating mechanical cues, which include physical forces and alterations in extracellular matrix mechanics, into biochemical signals, thus allowing cells to adapt to their physical environment. On the other hand, lamins A and C interact with chromatin through lamina-associated domains (LADs), which impact gene expression and regulation in a tissue-specific manner. The discovery that several genetic diseases are caused by mutations in the *LMNA* gene has resulted in a reassessment of the function of the nuclear lamina.

## 2. Laminopathies

Since the first description of *LMNA* mutations associated with Emery–Dreifuss muscular dystrophy (EDMD) through positional cloning in 1999 [4], the improvement of genetic strategies allowed the identification of other *LMNA* variants (reviewed by [5]). To date, more than 10 human clinical entities have been attributed to *LMNA* mutations. Collectively named laminopathies, they are commonly classified into four groups according to affected tissue: (i) adipose tissue, (ii) nervous system, (iii) accelerated ageing and (iv) striated muscles [6].

*LMNA* mutations cause partial lipodystrophy syndromes. Cao and Hegele identified *LMNA* mutations in a monogenic disorder: the Dunnigan-type familial partial lipodystrophy (FPLD) [7]. FPLD is characterized by loss of fat affecting the limbs and trunk from puberty; accumulation of fat in the neck and face; and predisposition to insulin resistance leading to complications such as glucose intolerance, dyslipidemia, high blood pressure, liver steatosis and increased risk for coronary heart disease. FPLD patients are born with normal fat distribution, but after the onset of puberty, there is regional loss of fat from the extremities associated with insulin resistance and, frequently, diabetes mellitus. All FPLD-causing mutations in patients are found within a small cluster of residues exposed on the surface of lamins A and C [8,9].

*LMNA* got a lot of attention in 2003, when two groups localized Hutchinson–Gilford progeria syndrome (HGPS), a premature ageing syndrome, to the *LMNA* locus [10,11]. In 1886, Jonathan Hutchinson first described what is now known as HGPS. The latter is an extremely rare genetic disorder associated with a characteristic aged appearance very early in life. Children with HGPS appear normal at birth but, within a year, begin to display effects of accelerated ageing. As they mature, the disorder causes children to age about a decade for every year of their life. On average, death occurs at the age of 13, with at least 90% of subjects dying from progressive atherosclerosis of the coronary and cerebral arteries, with tissues such as bone and skin also prominently affected. Interestingly, patients do not suffer from osteoporosis, cancer, increased abdominal fat, cataract or neurodegeneration, signs usually associated with ageing [12]. Recent studies have demonstrated that a microRNA (miR-9) expression prevents progerin accumulation in HGPS neural cells, which explains the absence of brain-associated disorders in HGPS patients [13]. HGPS is a sporadic autosomal dominant disease caused in nearly all cases by a de novo single-base substitution in codon 608 of exon 11 of *LMNA* [10,11]. The *LMNA* mutation creates a cryptic splice donor site, resulting in expression of a truncated pre-lamin A called “progerin” with a 50-amino acid internal deletion near the *C*-terminus. Pre-lamin A is normally farnesylated on the cysteine of a CaaX motif located at its *C*-terminus. The prenylated protein is then recognized by an endoprotease and cleaved 15 amino acids away, including the farnesylated cysteine. This cleavage site is deleted from progerin, and hence, it remains farnesylated. Even though no single mechanism has clearly emerged to explain the complex phenotype in HGPS, considerable data suggest that farnesylated progerin is the molecular culprit.

Other progeroid laminopathies have been described, including mandibuloacral dysplasia type A, discovered in 2002 and featuring an intermediate clinical phenotype between FPLD and HGPS. Moreover, the most severe laminopathy so far reported is Restricted dermopathy (RD), which is fatal soon after birth and is caused by accumulation of farnesylated prelamin A. RD should be considered a severe developmental disease mostly affecting skin, though the bone phenotype and molecular defect are almost similar to those of HGPS.

## 3. Striated Muscle Laminopathies

**Figure 1:**
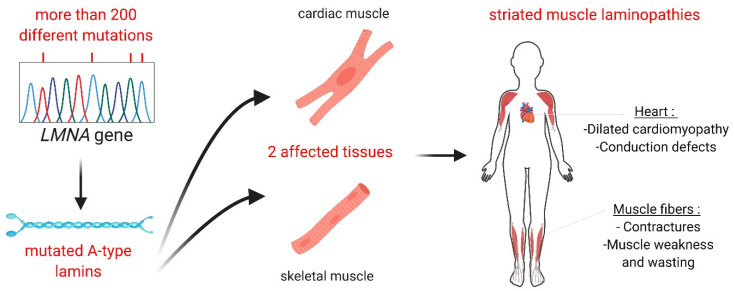
Striated muscle laminopathies: from genotype to phenotype. All along the *LMNA* gene, more than 200 different mutations have been related to striated muscle laminopathies (e.g., p.H222P, p.R190W and p.E65G mutations respectively lead to Emery–Dreifuss muscular dystrophy, limb girdle muscular dystrophy and dilated cardiomyopathy). These mutations in *LMNA* gene, encoding A-type lamins, affect both cardiac and skeletal muscle tissue. Patients with striated muscle laminopathies exhibit different clinical features such as contractures, muscle weakness and wasting, but they all share cardiac dysfunction as a common denominator.

Among striated muscle laminopathies, EDMD was first described by the study of a family suffering from early contractures, skeletal muscle weakness and cardiomyopathy [14]. The disease can be transmitted in both an autosomal and X-linked manner (mutations in the *EMD* gene, encoding emerin, which is a protein of the inner nuclear membrane interacting directly with lamins A and C) [15,16]. In EDMD, symptoms generally appear in the first decade of life. Contractures are the first clinical signs of disease and appear before muscle weakness and wasting, which is different from most other muscular dystrophies. Contractures affect the elbows, neck extensor muscles and Achilles tendons and prevent complete extension, leading to difficulties with posture and walking. The slowly progressive muscle weakness and wasting begins in a humero-peroneal distribution (biceps, triceps and peroneal), usually during the end of the second decade of life. Cardiac disease occurs in all cases of EDMD, and in most cases, symptoms begin at the end of the second decade, with no direct relationship to the severity of the skeletal muscle involvement. The initial presentation is usually atrioventricular conduction blockage, and over time, dilated cardiomyopathy develops. Sudden cardiac death is common in EDMD, and insertion of a defibrillator can be a lifesaving measure. Striated muscles laminopathies include also limb-girdle muscular dystrophies type 1 [16,17], *LMNA*-associated congenital muscular dystrophies [18] and isolated dilated cardiomyopathy with conduction defects [19]. These disorders share dilated cardiomyopathy as a common denominator, which has negative effects on health span and quality of life, leading in most cases to heart failure (Figure 1) (reviewed by [20]).

The mechanisms by which mutations in *LMNA,* the gene responsible for the autosomal form of EDMD, cause tissue-specific disorders are poorly understood. Research on striated muscle laminopathies in recent years has provided novel perspectives on the role of the nuclear lamina and has shed light on the pathological consequences of altered nuclear lamina [5]. Although progresses have been made to identify genes causing the disease and to decipher pathological mechanisms, there is currently no curative treatment for these diseases. An emerging body of work supports a new view of the nuclear lamins A and C as a node that integrates and transduces a range of signals during development and in terminally differentiated cells. Hence, beyond its classical barrier function, studies of the nuclear envelope are increasingly providing insights into basic aspects of cellular organization and function, providing novel insights into the pathogenesis of human disease. Dissecting these pathways would likely lead to the discovery of new mechanisms that control development and tissue-specific diseases. Since the discovery that *LMNA* mutations can cause striated muscle pathology, three mutually nonexclusive mechanisms have been hypothesized to underpin the pathogenesis: (1) impaired nuclear mechanoresistance via the LINC complex, or “mechanical hypothesis”; (2) alteration of lamin A/C-controlled intracellular signalling pathways, or “signalling hypothesis”; and (3) dysregulation of chromatin organization leading to gene expression alterations, or “chromatin hypothesis”.

## 4. NAD^+^: From Metabolism to Consumption

Altered nicotinamide adenine dinucleotide (NAD^+^) metabolism is associated with ageing and many pathological conditions, such as metabolic diseases, and disorders of the muscular system have recently been linked to the development of striated muscles laminopathies. This review focuses on the role played by NAD^+^ metabolism in striated muscles laminopathies, from bench to bedside.

NAD^+^ is a co-factor for several enzymatic reactions [21]. These processes lead to consumption of NAD^+^, resulting ultimately in a constant need to replenish cellular NAD^+^ stores. Thus, maintaining the balance between NAD^+^ degradation and biosynthesis is critical for cellular homeostasis. Skeletal muscle requires massive amounts of energy to sustain contractile function. Given that skeletal muscle energy stores are limited, altered NAD^+^ homeostasis could contribute to the pathogenesis of striated muscle diseases.

**Figure 2:**
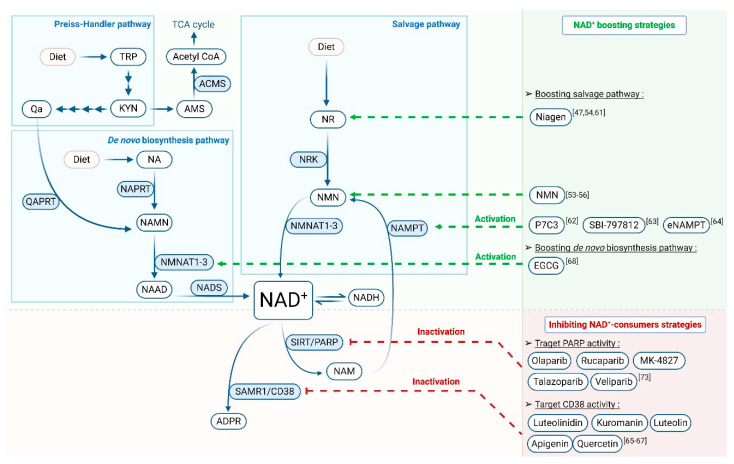
Clinical perspectives targeting altered nicotinamide adenine dinucleotide (NAD^+^) metabolism to treat striated muscle laminopathies: strategies allowing a raise in NAD^+^ synthesis or inhibiting its consumption have already proven their effects on NAD^+^ levels. Future studies may confirm the potential of these molecules in the treatment of laminopathies. ADPR: ADP-ribose, ACMS: Aminocarboxymuconic Semialdehyde, AMS: Aminomuconic Semialdehyde, CD38: Cluster of Differentiation 38, EGCG: Epigallocatechin Gallate, eNAMPT: extracellular Nicotinamide phosphoribosyltransferases, KYN: Kynurenic acid, MK-4827: Niraparib, NA: Nicotinic Acid, NAAD: Nicotinic Acid Adenine Dinucleotide, NAD: Nicotinamide Adenine Dinucleotide, NADH: reduced form of NAD, NADS: NAD Synthase, NAM: Nicotinamide, NAMN: Nicotinic Acid Mononucleotide, NAMPT: Nicotinamide Phosphoribosyltransferase, NAPRT: Nicotinic Acid Phosphoribosyltransferase, NMN: Nicotinamide Mononucleotide, NMNAT: Nicotinamide Mononucleotide Adenylyltransferase, NR: Nicotinamide Riboside, NRK: Nicotinamide Riboside Kinase, P7C3: Pool 7 Compound 3, PARP: Poly(ADP-Ribose)Polymerase, QA: Quinolinic Acid, TCA: Tricarboxylic Acid, TRP: Tryptophan, QAPRT: Quinolinic Acid Phosphoribosyltransferases, SAMR1: Sterile Alpha And TIR Motif Containing 1, and SIRT: Sirtuins.

In mammals, bearing in mind that only neurons are able to import NAD^+^ [22], tissues like striated muscles use independent intracellular pathways to produce NAD^+^. NAD^+^ can be synthesized from various ways (Figure 2).

De novo and Preiss–Handler pathways lead to NAD^+^ synthesis from tryptophan (Trp) and the vitamin B3 nicotinic acid (NA, Niacin), respectively, in the diet. De novo biosynthesis, which starts from the amino acid Trp, is considered a minor contributor to the total pool of NAD^+^ [23]. During a multi-step reaction called kynurenine pathway, Trp is firstly transformed in aminocarboxymuconic semialdehyde (ACMS) and is then converted into quinolinic acid (QA). The quinolinic acid phospho-ribosyl-transferase (QaPRT) generates the nicotinic acid mono-nucleotide (NAMN) from QA. In parallel, ACMS can be converted into aminomuconic semialdehyde (AMS) by the alpha-amino-beta-carboxy-muconate-semialdehyde decarboxylase (ACMSD). This reaction allows production of acetyl-CoA through tryptophan degradation, which is ultimately utilized in the tricarboxylic acid (TCA) cycle [24]. It is believed that the use of Trp for the TCA cycle or NAD^+^ synthesis is regulated by ACMS accumulation and, therefore, depends on ACMSD activity. Indeed, ACMSD overactivation prevents efficient NAD^+^ synthesis while low ACMSD activity allows efficient tryptophan-to-NAD^+^ conversion [24]. The Preiss–Handler pathway consists in transformation of NA into NAMN through the nicotinate phospho-ribosyl-transferase (NAPRT). Both de novo and the Preiss–Handler pathways end with nicotinamide mono-nucleotide adenylyl-transferease (NMNAT) enzymes that catalyse the NAMN conversion in nicotinic acid adenine dinucleotide (NAAD). Ultimately, NAD synthases (NADS) produce NAD^+^ from NAAD (reviewed in [25]). These pathways are key for NAD^+^ homeostasis. This is depicted by the human disease pellagra, which is caused by NAD^+^ deficiency subsequent to poor dietary intake of precursors. This disease is caused by deficiency of NAD^+^ and can be easily treated by providing vitamin B3 in the diet [26].

The salvage pathway is considered the most important pathway for maintaining intracellular NAD^+^ levels [27]. It is based on recycling of the degradation products generated by the enzymatic activities of NAD^+^ consumer enzymes such as Poly (ADP-Ribose) polymerase (PARPs), Sirtuins (SIRT) and Poly (Adenosine diphospho-ribose) (ADPR) synthases [28]. Initially, nicotinamide (NAM) released during NAD^+^ consumption reactions is recycled into nicotinamide mononucleotide (NMN) by the nicotinamide phosphoribosyl-transferases (NAMPT). The latter are the limiting enzymes of this pathway in converting NMN to NAD^+^ [29,30]. In parallel, nicotinamide riboside kinases (NRK1/2) transform nicotinamide riboside (NR) into NMN [31]. NMN is then converted in NAD^+^ by nicotinamide mononucleotide adenylyl-transferase 1, 2 and 3 (NMNAT 1, 2 and 3) that are respectively expressed in the nucleus, cytoplasm and mitochondria [32]. Supplementation of NR to cells or mice increases the levels of NAD^+^ and results in the activation of its downstream signalling cascades [31,32].

NAD^+^ was recently identified as an important co-substrate for different classes of enzymes, among which is PARPs [33]; 17 PARP enzymes have been discovered, 16 of which bind and cleave NAD^+^ to NAM and ADPR. They catalyse the attachment of one or more ADPR subunits to itself and to multiple acceptor proteins [34]. This reaction is called Poly (ADP-Ribosyl)ation (PARylation) [35]. PARylation is known to be attached to glutamate, aspartate, arginine and lysine residues of proteins by PARPs. PARylation is a reversible reaction, and ADPR units can be removed from acceptor proteins by a series of enzymes such as Poly (ADP-Ribose) glycohydrolase (PARG). The majority of cellular PARP activity is localized to the nucleus. Although PARylation was identified 50 years ago, surprisingly little is known about the molecular targets and which processes these specifically regulate. The covalent modification of proteins by PARylation is a biochemical response to deoxyribonucleic acid (DNA) damage induced by oxidation, alkylation and ionizing radiation. PARPs have widespread functions that are vital for cellular homeostasis [36]. The binding of PARP-1 to damaged DNA, including single-strand breaks (SSBs), double-strand breaks (DSBs) and base excision repair (BER), activates PARP-1 enzymatic activity [37,38]. Therefore, PARP-1 can function as a DNA damage sensor. With low levels of DNA damage, PARP-1 acts as a factor involved in DNA damage detection and repair. In contrast, with high levels of DNA damage, PARP-1 promotes cell death. PARP-1 interacts physically and functionally with various proteins involved in DNA repair pathways and may recruit the repair proteins to sites of DNA damage (e.g., X-ray Repair Cross Complementing 1 (XRCC-1) in BER and DNA-dependent protein kinase in DSB repair). Overall, PARPs contribute not only to DNA repair and cell death but also to carcinogenesis [39], metabolism [40], signalling [41], gene transcription [42] and ageing [43]. Further studies are needed for a better understanding of the role of each PARP isoform and their NAD^+^ consumption quantity.

The most-investigated enzyme that uses NAD^+^ as a co-substrate is Sirt1, a NAD^+^-dependent nuclear deacetylase that removes acetyl groups from lysine residues of a target protein, e.g., the tumour suppressor p53, the myocyte-specific enhancer factor 2 (MEF2), the forkhead box O1 (FoxO1) and peroxisome proliferator-activated receptor gamma co-activator 1-alpha (PGC-1α), all of which regulate transcriptional programs related to increased mitochondrial function [44]. Sirt1 activity is directly regulated by NAD^+^, raising the hypothesis that NAD^+^ acts as a metabolic sensor. For instance, both NAD^+^ levels and Sirt1 activity increase in mammalian tissues in response to energy/nutrient stresses such as exercise [45,46]. Sirt1 is part of the sirtuin family composed by seven homologs, which all are NAD^+^-dependent enzymes [47]. Ubiquitously expressed, Sirt1, 2 and 6 are located in both the nucleus and cytoplasm; Sirt4 is mitochondrial; Sirt7 is nuclear; and Sirt3 and 5 are found in the mitochondria, nucleus and cytoplasm [48]. Sirtuins have been initially considered as histone deacetylases and are now recognized to have several functions such as deacylase, demyristoylase, desuccinylase, deglutarylase, demalonylase, lipoamidase, decrotonylase and ADP-ribosyltransferase. Overall, sirtuins are NAD^+^-dependent deacetylases known to regulate key biological processes including chromatin regulation, cell cycle control, DNA repair and mitochondrial function [49].

Other NAD^+^ consumers such as cluster of differentiation 38 (CD38), and sterile α and TIR motif–containing 1 (SARM1) allow ADPR synthesis from NAD^+^ hydrolysis transported into the extracellular compartment [50]. Through their ADP-ribose synthase activity, CD38 and SARM1 are able to convert nicotinamide adenine dinucleotide phosphate (NADP) into nicotinic acid–adenine dinucleotide phosphate (NAADP) [51,52]. These cytosolic NAD^+^ metabolites are involved in calcium (Ca2^+^) signalling through regulation of ryanodine receptor (RyR) and transient receptor potential cation channel subfamily M member 2 (TRPM2) essential for contractile functions and several signal transduction events (reviewed by [53]).

## 5. Effect of NAD^+^ on Health

**Figure 3:**
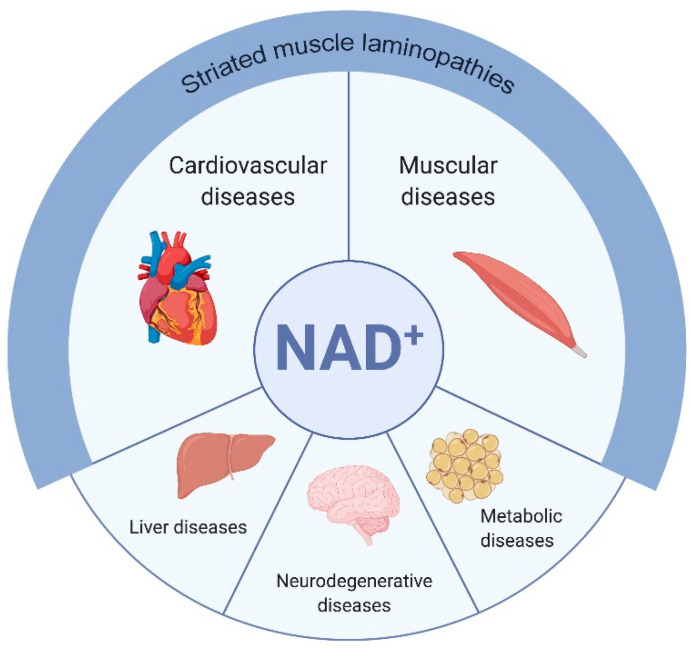
Central role of NAD^+^ in pathological conditions: impaired NAD^+^ levels are involved in the development of pathologies including cardiovascular, muscular, liver, neurodegenerative and metabolic diseases. Given that cardiac and skeletal muscles are the main affected tissues in striated muscle laminopathies, boosting NAD^+^ levels is expected to be an efficient treatment to improve patients health span.

The initial discovery that NAD^+^ is a master regulator of health [54,55,56] prompted investigation of NAD^+^ metabolism in disease models (Figure 3).

NAD^+^ signalling is known to protect from fatty liver diseases. Sirt1 is also an important regulator of metabolism, which regulates gluconeogenesis as a result of deacetylation of FoxO1 [57,58,59]; hence, maintaining NAD^+^ level is critical for liver function. Loss of Sirt1 from the liver accelerates the metabolic disturbances of a high-fat diet [60,61,62], while Sirt1 overexpression improves many of the metabolic consequences of obesity and diabetes [51,63,64,65]. Boosting NAD^+^ levels (see part 5) has been effective at preventing and treating obesity and alcoholic steatohepatitis and at improving glucose homeostasis as well as mitochondrial dysfunction. These approaches include the inhibition of NAD^+^ coenzymes such as PARPs [66,67] and CD38 [64]; the activation of nicotinamide N-methyltransferase; [68] and supplementation with NAD^+^ precursors, NR [32] or NMN [65].

Dysfunctional mitochondria are a hallmark of progressive neurodegenerative diseases, including Alzheimer’s disease (AD). AD is the most common form of dementia, affecting millions of people worldwide. AD is characterized by progressive cognitive impairment [69,70] resulting from synapse loss and neuronal death that are critical for learning and memory and emotional control. These lead to continuous decline in thinking, behavioural and social skills, altering a person’s ability to function independently. One of the identified contributing factors may be NAD^+^ depletion [71]. Since neurons have a high-energy demand, they are very sensitive to NAD^+^ depletion and impairment of ATP production [72]. A recent study demonstrated that supplementation with NR in a mouse model of AD can target several aspects of AD, setting the stage for future therapeutic perspectives in humans [73]. A deficit in the NAD^+^ pathway has been also linked to amyotrophic lateral sclerosis patients [74]. Wang and colleagues showed that NAMPT plays an essential role in mitochondrial bioenergetics, motor function and survival and identified the NAMPT-mediated NAD+ salvage pathway as a potential therapeutic target for degenerative diseases [74]. Along the same line of evidence, a NAMPT enzymatic activity enhancer, P7C3, was reported to prevent neuronal degeneration in amyotrophic lateral sclerosis and Parkinson’s disease models [75,76], strongly suggesting that NAMPT may play a key role in neurodegenerative diseases.

Neurodegeneration in the human retina can lead to Leber congenital amaurosis, a severe form of inherited photoreceptor-neuron degeneration resulting in congenital blindness. Mutations in *NMNAT1* have been recently proposed to be responsible for the disease, by lowering NAD^+^ levels in affected individuals [77]. In addition, NAD^+^ depletion was linked not only in Leber congenital amaurosis but also in a broad spectrum of retinal degenerations, opening up the possibility of using NAD^+^ intermediates as therapeutic agents [78]. Indeed, more recently, oral administration of a NAD^+^ precursor and/or gene therapy using the *Nmnat1* gene has been proven successful to prevent a neurodegenerative disease that causes vision loss [79].

## 6. “Breaking NAD” in Striated Muscle Laminopathies

At the crossing between mitochondria dysfunction, energetic defect, calcium dysregulation and oxidative stress, NAD^+^ appears as a relevant determinant in striated muscle laminopathies [80,81] (Figure 3).

### 6.1. NAD^+^ and Cardiomyopathy

The adult mammalian heart requires enormous amounts of energy to sustain contractile function. It has been hypothesized that altered NAD^+^ homeostasis could contribute to the pathogenesis of striated muscle diseases. We recently demonstrated that the NAD^+^ content was significantly decreased in the heart of *Lmna*^p.H222P/H222P^ mice, a model that recapitulates cardiomyopathy associated with *LMNA* mutation, as observed in patients [82,83]. Arimura et al. developed *Lmna* knock-in mice carrying the H222P mutation that was identified in the human *LMNA* gene in a family with typical EDMD. This mutation was also chosen because it putatively dramatically altered the coiled-coil organization of A-type lamins based on in silico analysis. They show that, at adulthood, *Lmna*^p.H222P/H222P^ mice develop muscular dystrophy and dilated cardiomyopathy associated with conduction defects, reminiscent of EDMD in human. This was the first *Lmna* mouse model mimicking human EDMD from gene mutation to clinical features. This alteration is responsible for the decrease in NAMPT expression, which was also observed in the patient’s heart with *LMNA* mutations [84]. NAMPT is known to be responsible for controlling NAD^+^ cellular content [65,85]. Therefore, decreased NAMPT expression is associated with cardiac disease while increased levels appear to be protective, corroborating the work from others [86,87]. Exogenous NAD^+^ supplementation restores intracellular NAD^+^ levels and counteracts hypertrophic cardiac response [87], confirming the key role of NAD^+^ in cardiac function. Recent studies have corroborated that NAD^+^ cell content may be an important determinant for cardiac function [88,89]. Therefore, we expanded the current knowledge by showing that abnormal NAD^+^ salvage causes heart failure and inherited cardiomyopathy associated with rare disease. All of these results using mouse and human models suggest the link between NAD^+^ cardiac content and cardiomyopathy.

### 6.2. NAD^+^ and Muscular Dystrophy

**Figure 4:**
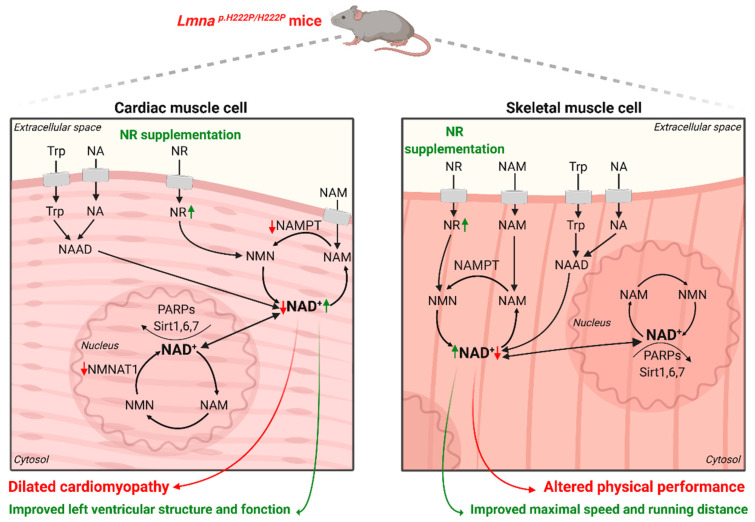
Main reported alterations in the NAD^+^ metabolism in a mouse model of striated muscle laminopathy: in cardiac tissue of *Lmna^p.H222P/H222P^* mice, an altered NAD^+^ salvage pathway has been observed with decreased NAMPT and NMNAT1 expression. Although many pathways allow maintenance of the NAD^+^ pool, a decrease in intracellular NAD^+^ levels has been described in both heart and skeletal muscled associated with dilated cardiomyopathy as well as altered physical performance. NR supplementation provides a rescue of striated muscles NAD^+^ content leading to a reduction of left ventricular dilatation, improved maximal speed and improved running distance.

Skeletal muscle is energetically expensive. Skeletal muscle cells take in glucose and store it as glycogen. When there is a need for more energy within muscle fibres, glycogen is converted to glucose that is used to generate adenosine triphosphate (ATP). As NAD^+^ is required for aerobic cellular metabolism, NAD^+^ localization to mitochondria is important for muscle function. NAD^+^ muscle content alteration and decreased NAMPT expression were observed in the skeletal muscle of *Lmna*^p.H222P/H222P^ mice [90]. Similar observations have been made for Duchenne muscular dystrophy [91]. A decrease in physical performance of *Lmna*^p.H222P/H222P^ mice was demonstrated with a decrease in the mean maximal speed and running distance [90]. These evidences suggest that NAMPT is important for muscle function. A recent study showed that deleting NAMPT in mice leads to a decreased NAD^+^ muscle content and a significant alteration in muscle mass and endurance [92]. This confirms that striated muscle NAD^+^ deficiency contributes directly to a decrease in muscle function, highlighting the role of NAD^+^ in skeletal muscle laminopathies (Figure 4).

## 7. Clinical Perspectives Targeting NAD^+^ Metabolism

Tissue concentrations of NAD^+^ decline in striated muscles during pathological conditions as described previously [89,90]. In order to compensate this deficit, strategies have been developed either to boost NAD^+^ synthesis or to reduce its consumption and, therefore, to palliate the pathological effects (Figure 2).

### 7.1. NAD^+^ Synthesis Boosting Strategies

To maintain NAD^+^ cellular content, several NAD^+^ precursors have shown beneficial effects on different pathologies [90,93], affecting either the heart [87,93,94] or the skeletal muscles [91,95]. NR is orally available for both mice and humans, making it the most studied therapeutic strategy [96]. In *Lmna*^p.H222P/H222P^ mice, NR supplementation (Niagen) has been shown to rescue striated muscle NAD^+^ content compared with untreated animals, ensuing an improvement of cardiac function and physical performance [84,90] (Figure 4). Niagen has been recently used in clinical trials for heart failure (ClinicalTrials.gov identifier: NCT03423342). Patients with striated muscle laminopathies could benefit from this therapy if the outcomes are positive. However, treatment of *Lmna*^p.H222P/H222P^ mice with another NAD^+^ precursor, NAM (NAMPT substrate), did not have beneficial effects on the cardiac NAD^+^ content and consequently showed no cardiac function [84]. This is potentially due to an inability to metabolize the intake of NAM by NAMPT and has therefore no beneficial effect on cardiac function [84,90]. In addition, boosting NAD^+^ levels with Niagen administration in a mice model of Duchenne muscular dystrophy improved cardiac and skeletal functions [91]. Recently, a reduced form of NR called 1-((2R,3R,4S,5R)-3,4-Dihydroxy-5-(hydroxymethyl)tetrahydrofuran-2-yl)4H-pyridine-3-carboxamide (NRH) has been identified as a bioavailable NAD^+^ precursor. In mammalian cells treated with NRH, intracellular NAD^+^ levels were significantly superior to cells treated with Niagen [97]. Given that NRH is orally bioavailable and does not degrade in mice plasma, NRH administration could be a potential treatment in order to boost NAD^+^ levels.

As a limiting enzyme of the NAD^+^ salvage pathway, NAMPT has been the preferential target of NAD^+^ boosting strategies. Different studies identified small molecules able to enhance NAMPT expression, such as P7C3, and ultimately NAD^+^ cellular content [98]. More recently, SBI-797812 has been identified as a NAMPT activator, which increased levels of NAD^+^ in the heart of treated mice [99].

Recently, a new target was identified for boosting NAD^+^ synthesis. As mentioned in the previous section, Trp could be degraded for both the TCA cycle and NAD^+^ synthesis. Given the way Trp is regulated by ACMSD activity, this enzyme represents a potential target in order to boost NAD^+^ synthesis. A selective inhibitor of human ACMSD has been discovered and is able to increase intracellular NAD^+^ levels [100]. Moreover, oral administration of ACMSD inhibitors in mice enhances NAD^+^ levels in the liver and kidney, which makes this pharmacological inhibition a potential candidate for clinical trials of striated muscle laminopathies treatment [101].

Increasing the extracellular level of NAMPT (eNAMPT) also appears to boost NAD^+^ synthesis. It has been shown that plasma levels of eNAMPT, which are carried in extracellular vesicles through blood circulation, significantly decline with age in both mice and humans. It has been proposed that supplementation with extracellular vesicle-contained eNAMPT could enhance NAD^+^ synthesis and improve health [102]. This needs to be tested in striated muscle laminopathies. Boosting cardiac NAD^+^ levels can also be achieved by NMN administration [89,93,103]. It has been shown that NMN administration prevents alteration of Sirt1 activity in mice cardiomyocytes, resulting in decreased susceptibility to apoptosis [104]. Metabolic diseases were also prevented by NMN supplementation [65]. These studies collectively indicate that NMN administration seems to be a promising treatment for cardiomyopathies. NMNATs are also interesting targets for raising NAD^+^ because of their contribution of both de novo and salvage pathways [22,105]. The compound has been reported to activate NMNATs, suggesting another potential way to increase NAD^+^ biosynthesis [106].

### 7.2. Inhibiting NAD^+^-Consumers Strategies

In addition to NAD^+^ boosting strategies, studies have shown that inhibiting NAD^+^ consumers allows increased NAD^+^ levels, resulting in protected tissue function. For instance, increased NAD^+^ levels in major organs including the liver, brain, muscle and heart were observed in CD38 knockout mice [107,108]. Experiments on these mice demonstrated that inhibiting CD38 protects hearts against ischemia and reperfusion. Moreover, glutathione levels were preserved, recovery of the left ventricular contractile function was increased and decreased myocyte enzyme release as well as infarct size were shown [109]. Therefore, inhibiting CD38 could be used as a valid strategy to restore NAD^+^ level. One strategy could consist in the use of flavonoids able to inhibit CD38 such as, luteolinidin, kuromanin luteolin, apigenin [110,111] and quercetin [112].

Although PARPs have vital functions for cellular homeostasis including DNA damage detection and repair, their overactivation can lead to NAD^+^ deficiency, resulting in cell death. Moreover, PARPs are major NAD^+^ consumers, which is why PARP inhibitors have also been developed. Some of them are used for cancers treatment such as olaparib, rucaparib, MK-4827 talazoparib and veliparib (reviewed by [113]). However, the link between these inhibitors and the NAD^+^ level has not been well studied. It has been shown that the microRNA overexpression, miR-149, in C2C12 myotubes inhibits PARP-2, leading to increased cellular NAD^+^ levels and SIRT-1 activity and leading to increased mitochondrial function and biogenesis [114]. Additional evidence implies that inhibition of PARP-2 can be cardioprotective. It has been shown that PARP-2 knockdown with siRNA protects neonatal rat cardiomyocytes from hypertrophy induced by angiotensin II via SIRT1 activation [115]. This could also lead to potential avenues for therapeutic strategies.

According to reviewed studies above, NAD^+^-consumer inhibitors are expected to be beneficial for striated muscle laminopathies as they are thought to improve cardiac or/and skeletal muscle (Figure 3).

## 8. Conclusions

Autosomal EDMD is caused by mutations in *LMNA* encoding nuclear A-type lamins. Although early initiation of treatments may delay progression and prolong the pretransplantation phase of the disease, more definitive therapies for EDMD await better mechanistic understanding of the molecular basis for this disease to develop specific treatments. To explain how mutations in proteins of the nuclear envelope can cause EDMD, it has been proposed that nuclear envelope abnormalities cause cellular fragility and a decrease in the mechanical resistance to stress. However, the mechanism through which mutated lamins cause striated muscle dysfunction remains obscure. NAD^+^ has emerged as a critical regulator of metabolism impacting human health through different NAD^+^ dependent signalling pathways. Studies have highlighted the role of NAD^+^ depletion in striated muscle laminopathies. This correlation would clearly identify the role of NAD^+^ on striated muscle laminopathies in order to develop therapeutic strategies. Different molecules have already proven their ability to increase NAD^+^ levels either by boosting its synthesis or by decreasing its degradation (Figure 2). In the future, elevation of NAD^+^ may offer viable therapeutic strategies to treat striated muscle laminopathies that remain uncured.
